# Association between Japanese community health workers’ willingness to continue service and two categories of motives: Altruistic and self-oriented

**DOI:** 10.1371/journal.pone.0220277

**Published:** 2021-10-06

**Authors:** Atsuko Taguchi, Hiroshi Murayama, Keiko Ono

**Affiliations:** 1 Division of Health Sciences, Department of Public Health Nursing, Tohoku University Graduate School of Medicine, Sendai, Japan; 2 Institute of Gerontology, The University of Tokyo, Tokyo, Japan; 3 Social Science Research Institute, International Christian University, Tokyo, Japan; University of Auckland, NEW ZEALAND

## Abstract

**Background:**

As population aging progresses, volunteers in health field are expected to play a key role in health promotion and disease prevention, which may improve community residents’ health and well-being and at the same time help slow the growth of healthcare cost. The objective of this study is to examine the effects of self-oriented motives and altruistic motives as explanatory factors for Japanese Community Health Workers (CHWs)’ desire to continue their service. Unraveling the relative effects of these two types of motivation on CHW retention may lead to policy and practical implications for recruiting, training, and supporting CHWs in Japan. Haddad (2007) observed that citizens in Japan generally have a sense of governmental and individual responsibility for dealing with social problems. Applying these insights to CHWs, we hypothesize that altruistic motives have more potent influence on volunteers’ willingness to continue to serve than self-oriented motives.

**Methods:**

Three cities in Shiga prefecture, Japan agreed to participate in the study. Anonymous, self-administered questionnaire was mailed to all CHWs who work in the three communities. The survey data were collected in March and April, 2013. A total of 417 questionnaires were mailed to CHWs, of which 346 were completed and returned (response rate 83.0%). Nine questionnaires missing response to the question concerning willingness to continue serving were removed from the analysis. The final analysis used 337 questionnaires (effective response rate 80.8%).

**Results:**

One hundred ninety-nine (59.1%) of the respondents answered the question about willingness to continue CHW affirmatively, and 138 (40.9%) negatively. Controlling for other relevant factors, those with self-oriented motives in serving as CHWs were more likely to state they are willing to continue to serve (OR:1.54, confidence interval 1.00–2.37) than those without such motives. Those with altruistic motives were also more likely to say they want to continue their service (OR 1.56, confidence interval 1.08–2.27) than those without such motives. Contrary to our hypothesis, the two motives, altruistic and self-oriented, were shown to have nearly equal degree of influence on respondents’ willingness to continue serving as CHWs.

**Conclusion:**

One practical implication of the research is that learning more about the twin motives, self-oriented and altruistic, of volunteers and tailoring the content of CHW training by municipal health professionals to address those motives may be beneficial.

## Introduction

Population aging is advancing in virtually all regions of the world today [1], but its advance is most pronounced in the affluent nations. The rapidly aging population in those countries presents a number of policy challenges, one of the most important of which is increased healthcare costs [2]. To help contain such cost increase, there has been a growing emphasis on health promotion and disease prevention to fight chronic, lifestyle related diseases [3,4] and to help older adults maintain their independence [5]. Health promotion and disease prevention measures targeting the elderly have been shown to not only mitigate the risk of common health issues such as falls but also to improve the socio-psychological well-being of the recipients of those measures [6].

It is increasingly difficult, however, for governments in industrialized nations to meet the needs of all citizens, given resource limitations [7], and health promotion and disease prevention is no exception. Regarding response to those challenges, there is a growing interest in the use of *volunteers* to provide health promotion and education services as a means of filling the gap between population needs and available resources [7,8]. In many nations there is already an established tradition of community health workers (CHWs), most of them uncompensated volunteers [9]. CHWs engage in health promotion and disease prevention by connecting residents to appropriate medical care available in the community, encouraging residents to use services, and facilitating access [9]. Community health workers delivering health information and advice to their neighbors could be a key to maintaining the physical and also the financial health of the nation [9].

Japan is among the most advanced aged (and aging) societies in the world; in 2017 its proportion of older adults (age 65 and older) was the highest in the world (27.1%; Li et al, 2019) [1]; that proportion is expected to reach roughly 40.0% by 2060 [10]. Consequently, health promotion volunteers in Japan, a variant of CHWs, increasingly focus both on supporting the health needs of the elderly and on educating all generations about prevention of disease and disability that tend to manifest later in life [11]. Though the CHW tradition in Japan dates back to around 1930, the priorities for CHW intervention have changed drastically since then, as society’s health needs have changed; the initial emphasis on prevention of infectious disease and improvement of nutrition have gradually been replaced by efforts to prevent chronic diseases associated with aging and to stave off frailty among the elderly. Roughly 80 percent of Japanese municipalities today recruit, train, and commission CHWs [12] to provide counseling and education about various health concerns to community residents.

Two major challenges in utilizing volunteers (including CHWs) in the social service and health sector are recruitment and retention [13,14]. The total number of volunteers serving in hospitals, for instance, is increasing, but at the same time premature dropping out is also on the rise [13]; high turnover means higher cost of recruitment and training and possible service disruption for the hiring organizations [13,15] as well as missed opportunity regarding full physical and psychological benefits from volunteer participation [16]. In the Japanese context, the overall decline in CHW volunteering has been attributed to rising female labor force participation, given that CHWs are predominantly home makers [17]. In addition, as average household size decreases, family members are responsible for a greater share of child care and elderly care duties, which leaves less time for CHW volunteer activity [18]. Delayed retirement, which is becoming more common in Japan these days, may also reduce the supply of senior volunteers [19]. Such societal changes are reflected by the decline in volunteer numbers in health-related volunteer institutions in Japan; the number of volunteers working in healthy eating promotion has decreased steadily since its peak in 1998 (220,000) [19], and the number of maternal health support volunteers decreased from 70,000 in 1993 to 42,000 in 2013 [20,21]. A 2017 survey of municipalities in Japan found that volunteers’ short term of service was a serious concern among officials [12]. Fewer volunteers could mean greater burden on those in service and could reduce willingness to participate [22,23]. Accurate knowledge of the factors influencing volunteers’ willingness to continue service is particularly important in Japan, where the available pool of new volunteers may shrink further in the future as a result of increased women’s labor force participation and further delays in the retirement age.

Davis et al. (2003) presents an elaborate model of the contribution of certain antecedents, including altruistic and self-oriented motivations, to initial volunteer decision to participate, and of how volunteers’ experiences mediate further involvement and continuation in service [24]. That model suggests that catering to volunteers’ motivations increases their satisfaction level and hence promotes their continued participation. Applying that model to the problem of CHW retention, it is clear that an understanding of how different motivations influence health volunteers to persist in their activities would be of use in efforts to promote volunteers’ active involvement and retention. Studies on volunteer retention have been inconclusive regarding the effect of motivation on volunteers’ desire to continue serving [14]. Moreover, few studies have examined CHW motivation to continue in service beyond the initial period of service. Regarding the Japanese case, we are not aware of any empirical study of CHW retention that examines volunteer motivation. Clearly, motivation is important here: in a survey of municipalities in Japan, many communities identified short volunteer tenure as a serious challenge [12]. Elucidation of the motivational and other factors that contribute to CHW willingness to continue to serve would be of use in the formulation of measures for maintaining volunteer morale and thus promoting both retention and new recruitment [25].

Psychological studies of volunteer motivation have identified two broad categories of motives for volunteer participation: self-oriented motives and altruistic motives [24–26]. Self-oriented motives for volunteering include desire for personal development and desire to learn, and altruistic motives include desire to help others and desire to fulfill social responsibility [25]. Initially, research focused on altruistic motives as the key to promoting volunteer participation [25,27]; more recently, research that assumes that motives for volunteer participation are multi-faceted, contextual, and complex have been dominant [24,26–28]. In the Japanese context, Sakurai [29] examined the association between volunteer motives for participation and (1) the characteristics of target activities and (2) volunteer attributes such as age and sex; altruistic motives were found to have greater influence than self-oriented motives on older volunteers’ willingness to continue in service. A similar finding is reported in a study conducted in Spain, which found that among senior volunteers “social” (altruistic) motivation exerted stronger influence on volunteer satisfaction than self-oriented motivation, but not among young volunteers [30].

In light of the above, the objective of this study is to examine the effect of self-oriented motives and altruistic motives [25] on Japanese CHWs’ desire to continue in service. Elucidation of the effects of these two types of motivation on CHW retention may be of use in policy formulation and may have practical implications for the recruiting, training, and support of CHWs in Japan. Haddad [31] observes that citizens in Japan generally have a sense that both government and the individual bear responsibility for dealing with social problems. Another reason to suspect that altruistic motives would have greater influence on CHWs is the aging of the volunteers in Japan. Altruistic motives were found to have greater influence than self-oriented motives on older volunteers’ willingness to continue in service in the existing literature [30]. Applying these insights to the problem of CHW retention, we hypothesize that altruistic motives have a greater influence than self-oriented motives on volunteer willingness to continue in service. We expect both altruistic and self-oriented motives to affect one’s willingness to continue service, but we hypothesize that the effect of altruistic motive would be greater.

## Data and methods

### Research areas and participants

The study was conducted in three communities (Kusatsu City, Ritto City, Yasu City) in southern Shiga Prefecture, Japan. Southern Shiga is a bedroom community half an hour by train from the city of Kyoto. Kusatsu, Ritto and Yasu had 126,000, 66,000 and 50,000 residents, respectively, in 2013. The proportion of elderly (age 65 and older) was lower than the national average: 18.0% in Kusatsu, 15.3% in Ritto, and 20.7% in Yasu.

To be commissioned as a CHW, an applicant must complete a minimum of 20 hours of training. In each city the public health center organizes and conducts CHW training, and offers additional training and support for in-service CHWs. Each city runs its own CHW program, but the three programs are quite similar. At the time of the study there were 180, 157 and 80 CHWs in Kusatsu, Ritto and Yasu, respectively.

### Methods

An anonymous, self-administered questionnaire was mailed to the CHWs. The survey data were collected in March and April, 2013. Initially, the research team met with public health center officials of the three cities to explain the objective of the study and to request their assistance in the distribution of the survey. The questionnaire was then sent to all 417 CHWs serving in the three cities that had agreed to participate in the study. Before distribution, a draft of the questionnaire was pretested with six CHWs who are serving in communities other than the study sites in the same prefecture. Based on the pretest results, questions were modified to improve their validity and to assist respondents’ comprehension.

This study was approved by the ethics review board of the Tokyo Metropolitan Institute of Gerontology. The research team explained the objectives of the research to the CHWs and city and public health officials in the three cities, who then consented to take part in the study. A letter was attached to each mailed questionnaire, explaining the purpose of the survey and clearly stating that (1) participation was voluntary and there were no negative consequences for non-participation, and that (2) data anonymity would be protected; the identity of the respondents would remain confidential. Filling out and returning the survey were taken as subject consent to participate.

### Measures

#### Willingness to continue to serve as CHW

CHW’s desire to continue serving was measured with a single Likert scale item; the participants were asked whether or not they agreed with the statement, “I would like to continue to serve as a CHW” (6: strongly agree, 5: agree, 4: somewhat agree, 3: somewhat disagree, 2: disagree, 1: strongly disagree). For ease of interpretation, the scale variable was converted into a dummy variable (1 if strongly agree, agree or somewhat agree, 0 if strongly disagree, disagree or somewhat disagree).

#### Altruistic and self-oriented motives for serving as CHWs

Studies of motivational factors related to becoming involved in CHW work and staying involved have mostly focused on altruistic and self-oriented motives [7,24–27,32]. Self-oriented motives include desire to acquire new knowledge and skills; altruistic motives include desire to help others and desire to contribute to society [7]. Previous studies have found some support for both self-oriented and altruistic motives as drivers for volunteer retention, and several studies report findings supporting self-oriented motives as drivers of retention [7,33,34]. One study conducted in Australia found a positive correlation between other-oriented motives and intention to continue volunteering, and a *negative* correlation between self-oriented motives and intention to continue [32].

To measure the respondents’ self-oriented and altruistic motives in serving as CHWs, the survey asked the respondents if they agreed with the following statements: “I do understand and appreciate the benefits for myself of serving as an CHW (such as gaining knowledge about health)” (self-oriented); and “I do understand and appreciate the benefits for community residents of CHW activities (such as CHWs being able to offer assistance in informal settings)” (altruistic). Responses were on a six-point scale ranging from 1: strongly disagree to 6: strongly agree.

#### Individual characteristics

Previous studies have examined entry into and persistence in volunteer activities [14]. Demographic characteristics which have been shown to correlate significantly with volunteer participation include age, sex, occupation, and race [7,34]. The questionnaire also asked respondent’s age, sex, educational attainment, work status, household economic situation, health status (self-rated), and household composition. The educational attainment item had three response categories: high school graduation (or less), junior college and other two-year tertiary educational institution graduation, four-year college graduation. The work status item had several response categories including full time and part time employment. The work status variable was converted into a dummy variable (1 if working full or part time or self-employed, 0 if not working or full time homemaker). As for household economic situation, respondents were asked to choose from 1: barely paying bills to 5: comfortable. Self-rated health responses were on a four-point scale (1: not healthy, 4: healthy).

#### Factors that might explain CHW motivation

Also assessed were years of service as CHW, level of motivation upon completion of initial training, and experience in a volunteer leadership role. Level of motivation responses were on a six-point scale (1: not at all motivated, 6: highly motivated).

### Analytical methods

First, bivariate analysis was conducted to examine the relationship between CHW willingness to continue to serve and each of the factors identified in the literature as possibly explaining such willingness. T test was applied to numeric variables, and Chi square test was conducted on categorical variables. Next, binary logistic regression analysis was conducted with desire to continue serving as the dependent variable (1 if willing to continue to serve, 0 if not). The main independent variables of interest were one self-oriented motive (understand and appreciate the benefits of serving to oneself) and one altruistic motive (understand and appreciate the benefits to local residents). Additional control variables were individual characteristics and several variables found in the literature to be relevant.

## Results

### Participant response rate and descriptive characteristics

A total of 417 questionnaires were mailed to the subjects, of which 346 were completed and returned (response rate 83.0%). Nine questionnaires with no response to the item concerning willingness to continue serving were removed before the analysis. The final analysis used 337 questionnaires (effective response rate 80.8%). One hundred ninety-nine of the respondents (59.1%) answered the question about willingness to continue CHW affirmatively, 138 (40.9%) negatively. The descriptive characteristics of the respondents are shown in Table 1. The average age of the volunteers was 62.2 years (standard deviation 7.2), and almost all were women (330 or 97.9%). The most common educational attainment was completion of middle school or high school graduation (n = 216, 64.1%), followed by completion of two-year tertiary education institution (n = 97, 28.8%), then completion of four-year college graduation (n = 20, 5.9%). A majority of the respondents (n = 200, 59.3%) were not employed. The mean self-rated health score was 3.2 (1: not healthy, 4: healthy) with standard deviation 0.7. Only a very small number of the participants (n = 12, 3.6%) lived by themselves; 320 (95%) lived with family member(s). The mean number of years of service as CHW was 8.5 years (standard deviation 6.0).

**Table 1:** Characteristics of the research participants. N = 337.

	Total n = 337 n (%)	Willingness to continue to serve as CHW		p-value
		No n = 138 n (%)	Yes n = 199 n (%)	
**Age** ^1)^	62.2±7.2	61.3±7.4	62.4±7.0	0.168
**Sex**				0.705^a^
Men	7 (2.1)	2 (1.4)	5 (2.5)	
Women	330 (97.9)	136 (98.6)	194 (97.5)	
**Educational attainment**				0.861
High school graduation or less	216 (64.1)	89 (64.5)	127 (63.8)	
Junior college or other two-year tertiary educational institution graduation	97 (28.8)	39 (28.3)	58 (29.1)	
Four-year college graduation	20 (5.9)	7 (5.1)	13 (6.5)	
**Work status**				0.238
Working	200 (59.3)	76 (55.1)	124 (62.3)	
Not working	133 (39.5)	76 (55.1)	73 (36.7)	
**Household economic situation** [range: 1–5]	3.5±0.8	2.9±0.8	2.7±0.8	0.012
**Self-rated health**^1)^ [range: 1–4]	3.2±0.7	3.0±0.7	3.3±0.6	<0.001
**Household composition**				0.373^a^
Living alone	12 (3.6)	3 (2.2)	9 (4.5)	
Living with family member(s)	320 (95.0)	131 (94.9)	189 (95.0)	
**Years of service as CHW** ^1)^	8.5±6.0	8.0±5.7	8.9±6.2	0.199
**Level of motivation upon completion of initial training**^1)^ [range: 1–6]	4.2±0.9	3.8±1.0	4.5±0.8	<0.001
**Experiences in CHW leadership role**				0.018
No experience	169 (50.1)	80 (58.0)	89 (44.7)	
Experience	162 (48.1)	56 (40.6)	106 (53.3)	
**Self-oriented motives for serving as CHW**^1)^ [range: 1–6]	4.2±0.9	3.9±0.9	4.4±0.7	<0.001
**Altruistic motives for serving as CHW**^1)^ [range: 1–6]	3.5±1.0	3.2±1.0	3.7±0.9	<0.001

^1)^ Mean±Standard deviation.

^a^: Fisher’s exact test.

no mark: Chi-square test.

### Analysis: Altruistic and self-oriented motives

Table 2 confirms that both self-oriented motive and altruistic motive had statistically significant impact on the volunteers’ willingness to continue the CHW activities. Controlling for other relevant factors, it was found that those with self-oriented motives for serving as CHWs were more likely than those without such motives to express willingness to continue to serve (OR:1.54, confidence interval 1.00–2.37). Contrary to our hypothesis, the effect of altruistic motives on respondent willingness to continue serving as CHW was comparable to that of self-oriented motives; the odds ratio was 1.56 (confidence interval 1.08–2.27).

**Table 2:** Binary logistic regression analysis of willingness to continue to serve as CHW. N = 303.

	B	OR	95%CI
**Age**	-0.02	0.98	(0.93–1.02)
**Sex** (ref = Men)	0.56	1.75	(0.27–11.19)
**Educational attainment** (ref = High school graduation or less)			
Junior college or other two-year tertiary educational institution graduation	0.01	1.01	(0.54–1.87)
Four-year college graduation	-0.09	0.92	(0.31–2.74)
**Household economic situation**	-0.18	0.84	(0.59–1.19)
**Self-rated health**	0.51	1.67	(1.09–2.55)
**Household composition** (ref = Living alone)	-1.20	0.30	(0.05–1.66)
**Years of service as CHW**	-0.03	0.97	(0.92–1.03)
**Level of motivation upon completion of initial training**	0.52	1.68	(1.20–2.35)
**Experience in CHW leadership role** (ref = no experiences)	0.62	1.86	(0.91–3.77)
**Self-oriented motives for serving as CHW** [range: 1–6]	0.43	1.54	(1.00–2.37)
**Altruistic motives for serving as CHW** [range: 1–6]	0.45	1.56	(1.08–2.27)

B: Regression Coefficient.

OR: Odds Ratio.

CI: Confidence Interval.

Self-rated health (OR: 1.67, confidence interval 1.09–2.55) and level of motivation upon completion of initial training (OR: 1.68, confidence interval 1.20–2.35) had positive and statistically significant effects on willingness to continue to serve, as hypothesized. No statistically significant association was found between desire to continue volunteering as CHW and age, sex, educational attainment, household economic situation, years of service as CHW, or experience in a CHW leadership role.

## Discussion

### The characteristics of the sample

Approximately 60% of the respondents expressed willingness to continue serving as CHWs. The sample was almost entirely all female (97.9%) and the average age was 62.2, significantly higher than the median age in the served communities. The majority of the study sample (59.3%) were not working at the time of the survey (“not working” includes full time homemakers). In terms of the above characteristics, the sample represents CHWs in Japan well; a national survey conducted earlier in Japan [12] found that CHWs are generally female and older (many volunteers are in their 60s). There is evidence that women tend to be more interested in and engaged with health issues than men, and that age has a positive relationship with interest in health [35]. Consequently, previous studies have also found that among health volunteers women greatly outnumber men, and that volunteers tend to be older [7,13,25].

### Willingness to continue serving: Self-oriented and altruistic motives

Controlling for other relevant factors, it was found that those who demonstrated understanding and appreciation of the personal benefits of serving as CHW were more likely to express willingness to continue to serve (OR:1.54, confidence interval 1.00–2.37). Our findings confirm those of several earlier studies [27,34,36]: both self-oriented motives and altruistic motives were related to the volunteer willingness to continue in CHW work. Haddad [31] makes the case that many citizens in Japan volunteer for activities that have a “close, embedded relationship with the local government.” [31]; she found that those who believe that the government, rather than individuals, must deal with social issues and those who consider volunteering to be a civic duty are more likely to serve as CHWs. Furthermore, Sakurai [29] found that homemakers and older adults who volunteer in Japan are motivated by altruism. In this study, we hypothesized that altruistic motives have a more robust impact on CHW retention than self-oriented motives in Japanese CHW organizations characterized by deep local ties; the empirical analysis, however, showed that the effect of self-oriented motives is as strong as that of altruistic motives. This could be explained by the transformation of Japanese society in the last few decades; information technology has been deeply integrated throughout Japan, providing higher quality of life on the one hand, but on the other hand resulting in in local community organizations becoming less relevant to the needs of the citizens [18]. Haddad [31] observed that Japanese citizens generally have a sense of governmental and individual responsibility for dealing with social problems. Our findings suggest that Japanese citizens’ sense of community responsibility may in fact be waning. Our findings confirm that self-oriented motives do affect CHWs motivation to continue serving in local community organizations; this is a significant contribution to the literature on CHW retention.

### Practical implications

The findings of this study can be usefully applied to inform CHW training. Extensive studies of CHW recruitment and training in a number of countries generally conclude that CHW training programs lack consistency in both length and curriculum content across administrative units [9]. In that regard, very few studies have evaluated the content of CHW training programs from the perspective of self-oriented and altruistic motives.

The findings here indicate that, in addition to altruistic motives, self-oriented motives play a significant role in volunteer retention. The findings are useful for CHW recruiting and training by municipal health professionals. One practical implication of that finding is that a better understanding of the two types of volunteer motivation, self-oriented and altruistic, and the tailoring of the content of CHW training to respond to those motives, may be beneficial for efforts to bolster volunteer retention. In Japan, the most important CHW activities are encouraging local residents to get health check-ups and conducting health education classes. In CHW training, volunteers acquire health knowledge, but the emphasis of training is primarily on the information needs of local residents, not those of the volunteers. However, many CHWs in Japan are older adults, which means that they often have health issues and concerns of their own; providing volunteers with training that also addresses their own health needs may increase the rate of volunteer retention; in that light, allowing volunteers to obtain advice from healthcare professionals concerning their own health in volunteer training might also be beneficial in terms of retention. Learning such things as how to interpret one’s own health check-up results and how to improve eating and exercise habits may fulfill volunteers’ self-oriented motives *and* at the same time may improve/enhance their performance in CHW activities.

Further research is needed to directly test this implication of our findings. In-depth interviews or focus group discussions, with both long serving CHWs, to gain insight into the reasons for their persistence; and those who have stopped serving, to determine their reasons for stopping, would be particularly valuable. If a correlation is found between continuation of service and the meeting of volunteers’ own health information/knowledge needs, that would constitute further support for our recommendations.

Analysis of the data revealed a negative correlation between volunteers’ self-oriented health and their expressions of willingness to continue serving. This suggests that the current level of CHW activities require volunteers to be healthy. Since it is well established in the literature that volunteering encourages social participation and motivates the elderly [37,38], there is clearly a need for a reexamination and modification of CHW activities to allow those with less than optimal health to continue to participate. In addition, if as expected Japan’s retirement age rises in the near future, many senior citizens may opt to work for pay rather than to volunteer; which would lead to more limited availability of senior volunteers. To address that challenge, volunteer organizations that rely on seniors may consider offering financial compensation for volunteer participation [19].

### Limitations

This study has several limitations. First, our research design is cross-sectional. To determine whether or not the volunteers actually continue in health volunteer work, a longitudinal research design is essential. Further collection of data among those who persist or drop out will shed more light on the factors that contribute to volunteer service continuation or discontinuation. Another limitation concerns measurement of volunteer motivation. In this survey, because of space limitation, a single question was used to assess volunteers’ self-oriented and altruistic motives. Further research is needed to evaluate and improve the reliability and validity of the question(s) and the scale. Finally, the study involved only a small number (n = 3) of health volunteer organizations, all located in the same region of a single prefecture in Japan.

## Conclusions

This study, explored the effects of self-oriented and altruistic motives on community health volunteers’ willingness to continue serving. Controlling for other relevant factors, it was found that those with self-oriented motives to serve as CHWs were more likely than those without such motives to express willingness to continue to serve (OR:1.54, confidence interval 1.00–2.37). Contrary to our hypothesis, the effect of altruistic motives on respondent willingness to continue in CHW work was as strong as that of self-oriented motives; the odds ratio was 1.56 (confidence interval 1.08–2.27).

One practical implication of the findings is that a better understanding of the self-oriented and altruistic motives of volunteers, and tailoring of CHW training by municipal health professionals content to address those motives, may contribute to efforts to enhance volunteer retention.

## Supporting information

S1 Dataset (SAV)

S1 File Questionnaire. (PDF)
