# Supplementary material for: Association between Japanese community health workers’ willingness to continue service and two categories of motives: Altruistic and self-oriented
Source: PLoS One. 2021 Oct 6;16(10):e0220277. doi: 10.1371/journal.pone.0220277 (PMC8494310; doi:10.1371/journal.pone.0220277)
Supplement: S1 File — (PDF) [file pone.0220277.s002.pdf]

健康推進員組織のアンケート調査
Questionnaire for Community Health Worker (CHW)

Q１．あなたは推進員になって何年目になりますか。
How many years have you been serving as a CHW?

（　　　　　　）年目(years)

Q２．あなたが認識している推進員活動の目的や意義について、あなたご自身がお感じになっていることに、最も近いものを選んで○をつけて下さい。**（○は1つずつ）**
On each of the goals and benefits of Community Health Worker activities that you may be aware of (shown below), which of the following is closest to your view? Choose only one among the response options.

	非常にそう思う	そう思う	まあそう思う	あまりそう思わない	そう思わない	全くそう思わない
１）自分にとっての推進員活動の意義（健康に関する知識が得られる、等）を、理解している<br>I do understand and appreciate the benefits for myself of serving as an CHW (such as gaining knowledge about health)	**6**	**5**	**4**	**3**	**2**	**1**
２）地域住民にとっての推進員活動の意義（住民に身近な立場で相談にのれる、等）を、理解している<br>I do understand and appreciate the benefits for community residents of CHW activities (such as CHWs being able to offer assistance in informal settings)	**6**	**5**	**4**	**3**	**2**	**1**

6: Strongly agree, 5: Agree, 4: Somewhat agree, 3: Somewhat disagree, 2: Disagree, 1: Strong disagree

Q３．**養成講座修了時**に、あなたは推進員活動に対してどの程度やる気を感じていましたか。**（○は1つ）**
At the end of the initial training course, how motivated were you to start engaging in CHW activities?

1．非常にやる気を感じていた
Highly motivated

2．やる気を感じていた
Motivated

2．まあやる気を感じていた
Somewhat motivated

3．あまりやる気を感じていなかった
Not very motivated

5．やる気を感じていなかった
Not motivated

6．全くやる気を感じていなかった
Not at all motivated

Q４．あなたは推進員活動をこれからも続けたいと思いますか。**（○は1つ）**
Do you wish to continue serving as a CHW? Choose one of the following response options.

1．全くそう思わない
Strongly disagree

2．そう思わない
Disagree

3．あまりそう思わない
Somewhat disagree

4．まあそう思う
Somewhat agree

5．そう思う
Agree

6．非常にそう思う
Strongly agree

VI. あなたご自身のことについてお尋ねします。
A few questions about yourself

Q１．あなたの性別をお答え下さい。**（○は1つ）**
Please indicate your gender.

１．女性 Female
２．男性 Male

Q２．あなたの年齢をお答え下さい。
Please indicate your age.

（　　　　　　）歳 years

Q３．現在、あなたが同居しているご家族の世帯構成は、次のどれにあてはまりますか。**（○は1つ）**
Which of the following best describes your current living arrangement? Choose one.

１．一人暮らし
Living alone

２．夫婦のみ
With a spouse

３．核家族または２世代家族
Nuclear family or two-generation family

４．３世代家族
Three-generation family

５．その他 Other（　　　　　　　　　　　　　　）

Q４　あなたの現在の主な職業または就業形態をお答え下さい。**（○は1つ）**
Which of the following best describes your work status? Choose one.

１．常勤
Working full time

２．非常勤（パート・アルバイト）
Working part time

３．自営業・家族従業員
Self-employed or work in family business

４．農林漁業
Working in agriculture, forestry or fisheries

５．無職（主婦含む）
Unemployed (including homemaker)

６．その他（　　　　　）
Other

Q５．あなたの世帯の、今の暮らし向きはいかがですか。**（○は１つ）**
How would you describe the current economic situation of your household? Choose one.

１．ゆとりがある
Comfortable

２．どちらかといえばゆとりがある
Somewhat comfortable

３．ふつう
Average

４．どちらかといえば苦しい
Just getting by

５．苦しい
Barely paying bills

Q６．あなたの最終学歴をお答え下さい。**（○は1つ）**
What is the highest level of education you have completed? Choose one.

１．中学卒業 Junior high school diploma
２．高校卒業　High school diploma
３．短大・高専卒業　Junior college degree
４．専門学校卒業 Other two-year tertiary educational institution
５．大学卒業以上 Four-year college degree
６．その他 Other（　　　　　　　　　　　　　　　　　　）

Q７．あなたの健康状態はいかがですか。**（○は1つ）**
How would you characterize your own health today? Choose one.

１．健康だと思う
Healthy

２．どちらかといえば健康だと思う
Mostly healthy

３．どちらかといえば健康ではないと思う
Not very healthy

４．健康ではないと思う
Not healthy
